# Modulation of Neural Microcircuits That Control Complex Dynamics in Olfactory Networks

**DOI:** 10.3389/fncel.2021.662184

**Published:** 2021-06-22

**Authors:** Zhenbo Huang, Roberta Tatti, Ashley M. Loeven, Daniel R. Landi Conde, Debra Ann Fadool

**Affiliations:** ^1^Program in Neuroscience, Florida State University, Tallahassee, FL, United States; ^2^Cell and Molecular Biology Program, Department of Biological Science, Florida State University, Tallahassee, FL, United States; ^3^Institute of Molecular Biophysics, Florida State University, Tallahassee, FL, United States

**Keywords:** olfactory bulb, cholecystokinin, leptin, glucagon-like peptide 1, GLP-1, acetylcholine, mitral cell, CCK

## Abstract

Neuromodulation influences neuronal processing, conferring neuronal circuits the flexibility to integrate sensory inputs with behavioral states and the ability to adapt to a continuously changing environment. In this original research report, we broadly discuss the basis of neuromodulation that is known to regulate intrinsic firing activity, synaptic communication, and voltage-dependent channels in the olfactory bulb. Because the olfactory system is positioned to integrate sensory inputs with information regarding the internal chemical and behavioral state of an animal, how olfactory information is modulated provides flexibility in coding and behavioral output. Herein we discuss how neuronal microcircuits control complex dynamics of the olfactory networks by homing in on a special class of local interneurons as an example. While receptors for neuromodulation and metabolic peptides are widely expressed in the olfactory circuitry, centrifugal serotonergic and cholinergic inputs modulate glomerular activity and are involved in odor investigation and odor-dependent learning. Little is known about how metabolic peptides and neuromodulators control specific neuronal subpopulations. There is a microcircuit between mitral cells and interneurons that is comprised of deep-short-axon cells in the granule cell layer. These local interneurons express pre-pro-glucagon (PPG) and regulate mitral cell activity, but it is unknown what initiates this type of regulation. Our study investigates the means by which PPG neurons could be recruited by classical neuromodulators and hormonal peptides. We found that two gut hormones, leptin and cholecystokinin, differentially modulate PPG neurons. Cholecystokinin reduces or increases spike frequency, suggesting a heterogeneous signaling pathway in different PPG neurons, while leptin does not affect PPG neuronal firing. Acetylcholine modulates PPG neurons by increasing the spike frequency and eliciting bursts of action potentials, while serotonin does not affect PPG neuron excitability. The mechanisms behind this diverse modulation are not known, however, these results clearly indicate a complex interplay of metabolic signaling molecules and neuromodulators that may fine-tune neuronal microcircuits.

## Introduction

**Figure 1:**
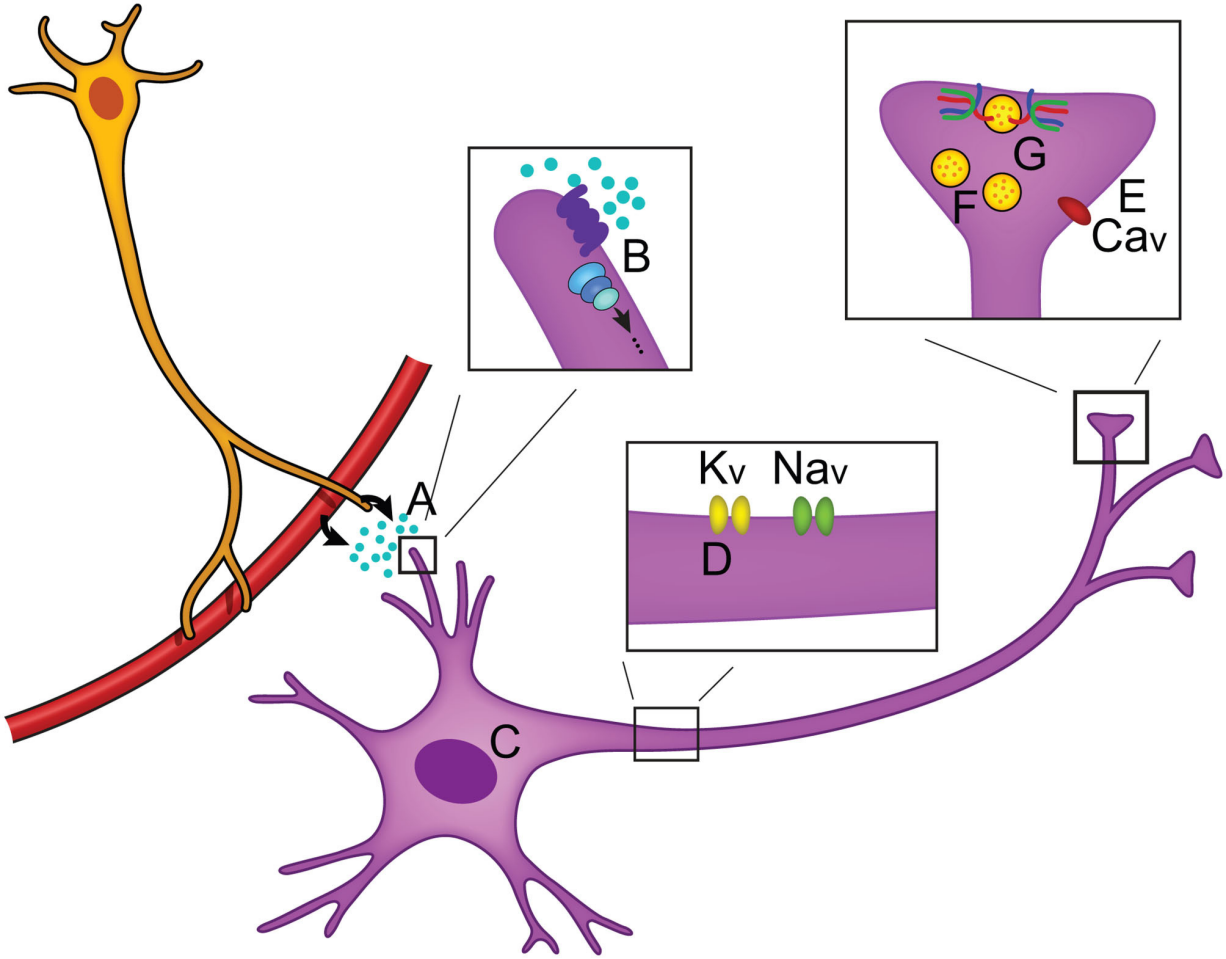
Overview of neuromodulation. **(A)** Neuromodulators can be released locally or circulated from the periphery and often bind to GPCRs **(B)** to activate intracellular signaling cascades. Intracellular effects include changes in **(C)** gene expression, **(D)** axonal sodium and potassium ion channel activity, and **(E)** changes in calcium ion channel activity (Zhou et al., 2007; Gray et al., 2017; Williams et al., 2018). **(F)** Neuronal activity can be modulated by changing the number of vesicles that are in the reserve pool, readily releasable pool, or docked at the synaptic terminal, thus changing the likelihood of neurotransmitter release (Rosenmund and Stevens, 1996; Logsdon et al., 2006; Taschenberger et al., 2016). **(G)** Modulation of the SNARE complex activity has been found to regulate neurotransmitter release (Sakisaka et al., 2008; Liu et al., 2018).

When neurotransmitters are released from synaptic termini, information transfer takes place. This simple mechanism is the foundation of how we make decisions, learn, process emotions, or use our senses to interpret and navigate our external environments. By changing these parameters, or even factors regulating the likelihood of neurotransmitter release, our global behavioral state can impact how information is processed. This is the field of neuromodulation, the means by which our physiological state dynamically influences aspects of synaptic activity, neural excitability, and gene expression (Florey, 1967). Neuromodulatory mechanisms are numerous and target different aspects of neuronal activity to produce diverse effects, but ultimately each fine-tunes the information being transferred (Figure 1). The largest group of neuromodulators bind to GPCRs and activate G proteins that initiate intracellular signaling cascades via second messengers (Chen et al., 2006; Newton et al., 2016; Byczkowicz et al., 2019; Moro et al., 2020). Subsequent to GPCR binding, effects include changes in gene expression (Fukuchi et al., 2015), ion channel properties that impact action potential propagation (Burke and Bender, 2019), and even interaction of G_βγ_ with the soluble NSF attachment protein REceptor, or SNARE complex, inhibiting neurotransmitter release (Zurawski et al., 2019; Hamm and Alford, 2020). Useful *in vivo* techniques are emerging to study neuromodulatory signaling including a mouse model allowing for real time cAMP visualization (Kim et al., 2014; Wu et al., 2015; Muntean et al., 2018), and fluorescent biosensors for several neurotransmitters (Leopold et al., 2019).

**Figure 2:**
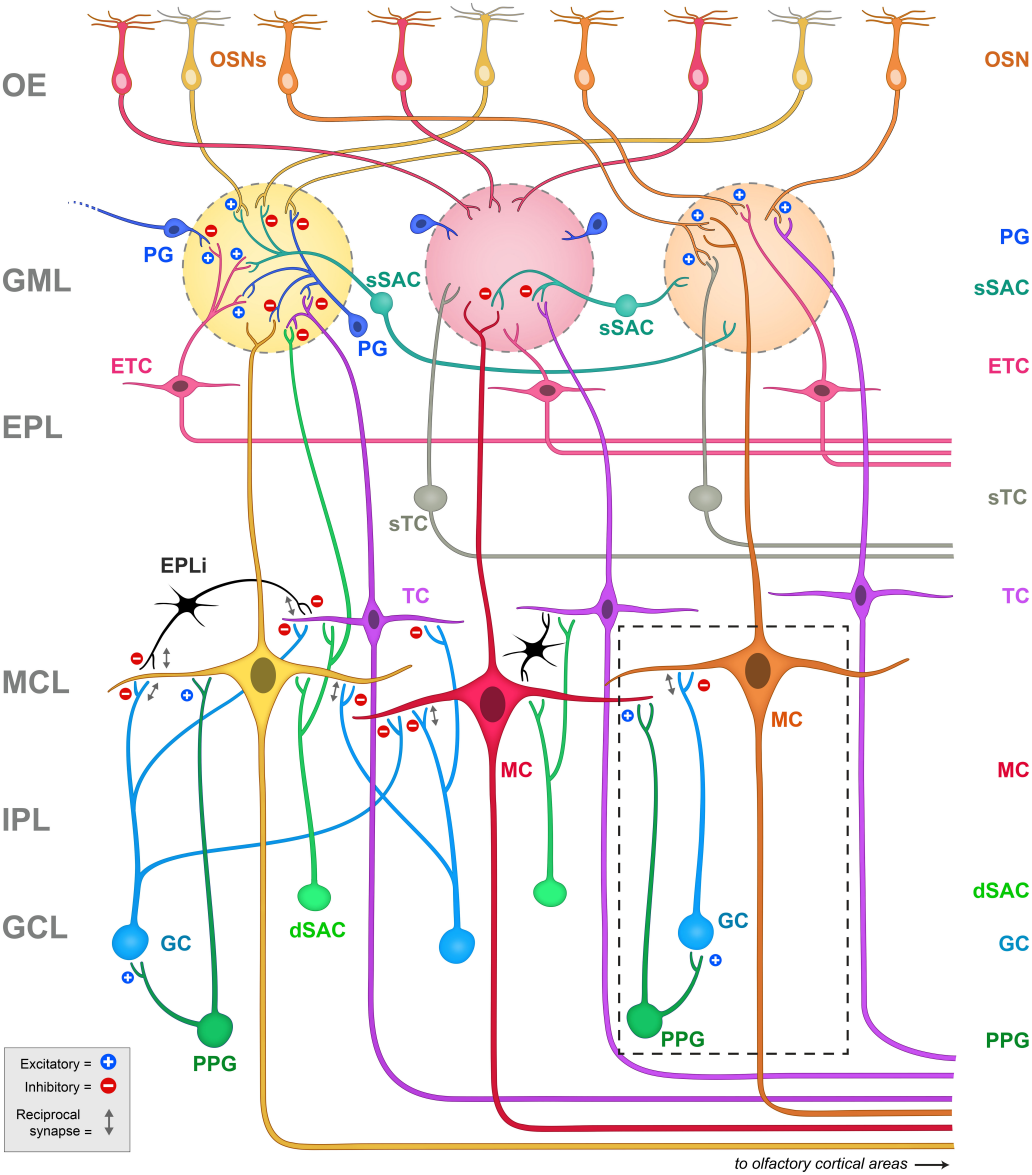
Schematic representation of the principal projection neurons and interneurons in the olfactory bulb, including the synapses between them. Blue plus signs (+) represent excitatory synapses, while red minus signs (-) represent inhibitory synapses. Reciprocal synapses are indicated by a gray double arrow. Note the highly laminated organization of the region that has been amendable for physiological and anatomical studies since first explored by Ramon y Cajal. The glomeruli are considered the first sensory processing station along the olfactory pathway where the olfactory signal is transferred from OSNs to glutamatergic output neurons, called mitral (MC) and tufted (TC) cells (Nagayama et al., 2014). Mitral and tufted cells' axons project to the olfactory cortex and higher order cortical structures conveying the information to the central nervous system (Ghosh et al., 2011; Nagayama et al., 2014). Both mitral and tufted cells (M/TCs) send an apical dendrite into a defined glomerulus, where they establish reciprocal synapses with OSNs and with a heterogeneous population of juxtaglomerular (JG) cells that include periglomerular (PG) neurons, external tufted cells (ETCs) and short axon cells (SACs). ETC have been shown to form an intrabulbar network of isofunctional columns and as such do not project out the of OB (Belluscio et al., 2002; Lodovichi et al., 2003; Zhou and Belluscio, 2008). MCs lateral dendrites form reciprocal synapses with granule cell (GC) dendrites whose cell bodies are located in the granule cell layer (GCL). The GCL also contains other types of neurons including deep short axon cells (dSACs) and several non-GCs populations (Ramon y Cajal, 1911; Price and Powell, 1970a; Schneider and Macrides, 1978). Very little is known regarding the role of these non-GC neurons and the modulatory afferents they receive. Dashed box = schematic representation of the PPG>MC>GC microcircuit. Note that the PPG neuron (forest green) is a subset of the dSAC variety (kelly green) located within the GCL. It has excitatory synaptic connections with both the GC and the MC (blue plus signs), following which, the GC has a classical dendro-dendritic reciprocal synapse onto MCs (gray double arrow) where it can exert inhibition (red minus sign). OE, olfactory epithelium; GML, glomerular layer; EPL, external plexiform layer; MCL, mitral cell layer; IPL, internal plexiform layer; GCL, granule cell layer; OSNs, olfactory sensory neurons; PG, periglomerular cell; sSAC, superficial short axon cell; ETC, external tufted cell; sTC, superficial tufted cell; TC, tufted cell; MC, mitral cell; dSAC, deep short axon cell; GC, granule cell; PPG, preproglucagon neuron; EPLi, interneuron of the external plexiform layer.

We have been exploring neuromodulation and information processing within the olfactory bulb, the first relay center for olfactory signals. Our intention is to understand how the physiological states of satiety, fasting, or over-nutrition can perturb or modulate transmission of olfactory information that ultimately can change eating behaviors (Palouzier-Paulignan et al., 2012; Julliard et al., 2017; Kolling and Fadool, 2020). In this topical issue, several authors have presented the functional synaptic activities of the known olfactory bulb circuitry (Ackels et al., 2020; Egger and Diamond, 2020; Imamura et al., 2020), so readers are directed to those works as an overview of the comprehensive neural circuit. The olfactory field is rich with investigations of synaptic interactions that drive an understanding of anatomical relationships and physiological mechanisms that ultimately modulate mitral/tufted (M/TC) cell output and subsequent olfactory behavior or detection (i.e., Shepherd, 1972; Jahr and Nicoll, 1980; Orona et al., 1984; Ezeh et al., 1993; Isaacson and Strowbridge, 1998; Aungst et al., 2003; Hayar et al., 2004a,b; Hayar et al., 2005; Zhou and Belluscio, 2008; Abraham et al., 2010; Huang et al., 2013; Banerjee et al., 2015; Najac et al., 2015; Liu et al., 2016; Burton, 2017; Pressler and Strowbridge, 2017; Harvey and Heinbockel, 2018; Jones et al., 2020). Herein, as schematized in Figure 2, we wish to home in on interneurons within the olfactory bulb that can provide neuromodulation of contrast and gain of the mitral/tufted (M/TC) cell output. These interneurons include those within the glomerular layer (GML), those within the external plexiform layer (EPL), and those centrally in the granule cell layer (GCL). It is also important to note that olfactory circuits do not solely rely on a linear feedforward transmission to interpret the external chemical environment - higher processing centers of the brain also present reciprocal connections with the olfactory bulb to modulate activity. These reciprocal connections mainly target GABAergic interneurons to modulate contrast and gain of M/TC output (Price and Powell, 1970b; Engel et al., 2001; Arevian et al., 2008; Fukunaga et al., 2012; Nagayama et al., 2014; Padmanabhan et al., 2018).

Due to the complexity of the neurolamina and diversity of the interneurons in the olfactory bulb, discovery of the mechanisms of neuromodulation of the olfactory output remains an ongoing process. This is particularly true for the largest neurolamina of the bulb, the granule cell layer (GCL), where much is known regarding the inhibitory network of granule cells (GC), yet the heterogeneity of non-GCs types in this region (Ramon y Cajal, 1911; Price and Powell, 1970a; Schneider and Macrides, 1978; Nagayama et al., 2014) does not afford a clear or completed picture of synaptic communication. A population of pre-proglucagon (PPG) neurons in the GCL has been discovered (Merchenthaler et al., 1999; Thiebaud et al., 2016) to project axons to the internal plexiform layer (IPL) and the mitral cell layer (MCL), and are speculated to release glucagon-like peptide 1, or GLP-1 (Thiebaud et al., 2016, 2019). The PPG neurons are a specialized type of deep short-axon cell (dSAC) (Eyre et al., 2008) and present stellate dendrites with abundant dendritic spines (Thiebaud et al., 2016, 2019; Burton et al., 2017). Stimulating PPG neurons can produce an excitatory or an inhibitory response on MCs due to a multi-synaptic interaction: PPG neurons form dendrodendritic synapses with MCs (PPG-MC) and with granule cells (PPG-GC). These three cell types therefore form a PPG neuron>MC>GC microcircuit (Figure 2, dashed box). Both synapses are usually excitatory, but stimulating GCs results in an inhibition of MCs through the release of GABA (Thiebaud et al., 2019). The functional significance of the microcircuit they hence establish, as a unique excitatory class of glutaminergic interneuron, remains incompletely known. Previous research on PPG neurons in the nucleus tractus solitarius (NTS) has shown that these neurons could be modulated by metabolic-related hormones such as cholecystokinin (CCK) (Hisadome et al., 2011) and leptin (Hisadome et al., 2010). These NTS PPG neurons have been suggested to provide a link between the energy state of an individual and their response to stress (Maniscalco et al., 2015). A negative energy balance induced by overnight fast was shown to block neural and behavioral responses to acute stress through inhibiting the activity of the NTS PPG neurons (Maniscalco et al., 2015). By comparison, PPG neurons in the olfactory bulb could act as a link between the individual's energy/nutritional state and their olfactory response. The expression of a variety of metabolic hormones such as ghrelin, orexins, leptin, insulin, CCK and their receptors (Palouzier-Paulignan et al., 2012) would allow the olfactory bulb to detect metabolic state while simultaneously modulating olfactory information processing.

Gut peptides such as GLP-1, CCK and leptin have been well-demonstrated to modulate olfactory circuit dynamics and could serve as plausible neuromodulators of PPG neurons (Ravel et al., 1990; Lemaire et al., 1994a,b; Prud'homme et al., 2009; Palouzier-Paulignan et al., 2012; Ma et al., 2013; Thiebaud et al., 2016, 2019; Sun et al., 2019) (see Table 1). Indeed, in the nucleus of the solitary tract, GLP-1-expressing neurons are modulated by CCK and leptin (Hisadome et al., 2010, 2011). It is not known whether the analogous PPG neurons in the olfactory bulb are also modulated by leptin and CCK. CCK was first reported in the gastrointestinal tract and later in the CNS (Vanderhaeghen et al., 1975). It represents the most abundant neuropeptide in the CNS, being found in the amygdala, cerebral cortex, hypothalamus, and olfactory system. Specifically within the olfactory system, CCK is expressed in the olfactory bulb, the olfactory tubercle and the piriform cortex (Beinfeld et al., 1981; Dupont et al., 1982; Ekstrand et al., 2001; Gutiérrez-Mecinas et al., 2005). Within the olfactory bulb, CCK immunoreactivity is detected in the superficial tufted cells and in the IPL (Marks et al., 2006; Kosaka and Kosaka, 2007) while the CCK receptors are located in the IPL, juxtaglomerular and MCL (Mercer and Beart, 1997). Leptin, alternatively, is produced by peripheral adipocytes and is involved in the regulation of body weight and food intake depending upon the nutritional state (Friedman and Halaas, 1998; Baly et al., 2007). Several studies support peripheral and central production of leptin (Morash et al., 1999). Leptin is capable of crossing the blood brain barrier using a saturable receptor-mediated mechanism (Banks, 2001). Leptin receptors are found in the central nervous system including the hypothalamus and the olfactory bulb (Guan et al., 1997; Elmquist et al., 1998). Fasting increases the transcription of leptin mRNA. Specifically within the olfactory system, leptin receptors have been shown to modulate spontaneous and odor-evoked electric activity in olfactory sensory neurons and to decrease the spontaneous firing of MCs (Baly et al., 2007; Savigner et al., 2009). *In vivo* experiments indicate that leptin inhibits odor-evoked oscillations (Sun et al., 2019) and decreases olfactory sensitivity (Julliard et al., 2007; Alkam et al., 2011; Sun et al., 2019).

**Table 1:** Overview of gut peptides and hormones that modulate olfaction.

**Neuromodulator**	**Source**	**Receptor**	**Localization**	**Cellular effects**	**Behavioral effects**	**References**
Leptin	White adipose tissue Leptin mRNA/protein found in brain tissue	Ob-R, mutant receptor protein in *db/db* mice	Olfactory sensory neurons Granule cell layer Mitral cell layer	Decrease signal-to-noise ratio of olfactory sensory neurons Inhibit granule cells Inhibit mitral/tufted cells, decrease Ca^2+^ response	Decreased performance in go, no-go discrimination task, slow reaction time, decrease olfactory sensitivity	Tartaglia et al., 1995; Lee et al., 1996; Guan et al., 1997; Elmquist et al., 1998; Shioda et al., 1998; Baly et al., 2007; Julliard et al., 2007; Prud'homme et al., 2009; Savigner et al., 2009; Palouzier-Paulignan et al., 2012; Sun et al., 2019
Cholecystokinin	Intestine Widespread in brain All bulb layers except for olfactory nerve layer, especially external tufted cells and superficial tufted cells of internal plexiform layer	CCK 1R CCK 2R	Internal granular layer Olfactory bulb, mitral cells	Excitation and suppression of mitral cell firing CCK 2R activation enhances inhibition of mitral/tufted cells	CCK 1R and CCK 2R modulate olfactory recognition in a social memory task via different pathways	Vanderhaeghen et al., 1975; Beinfeld et al., 1981; Zarbin et al., 1983; Crawley, 1985; Seroogy et al., 1985; Schiffmann and Vanderhaeghen, 1991; Lemaire et al., 1994a,b; Mercer and Beart, 1997; Mercer et al., 2000; Gutiérrez-Mecinas et al., 2005; Marks et al., 2006; Kosaka and Kosaka, 2007; Ma et al., 2013; Liu and Liu, 2018

*Ob-R, leptin receptor; db/db, leptin receptor mutant mouse model; CCK, cholecystokinin*.

Although it is not known if CCK or leptin have the capacity to modulate neural activity of the olfactory PPG neurons, as mentioned above, the interneurons in the GCL of the olfactory bulb additionally receive multiple centrifugal projections from higher brain areas including serotonergic, noradrenergic, cholinergic, and cortical feedback fibers. These centrifugal projections are believed to modulate olfactory information processing depending upon an animals' metabolic state.

Afferent serotonergic fibers that originate from the dorsal and medial raphe nuclei innervate all layers of the olfactory bulb, and thus the transmitter could serve as a neuromodulator of PPG neurons. One important mechanism underlying the ability to achieve diverse serotonergic modulation in the olfactory bulb is the broad expression of serotonin (5-HT) receptor subtypes (I to III). Indeed both *in vitro* and *in vivo* studies have shown diverse effects on different targets in the olfactory bulb by serotonergic modulation (Hardy et al., 2005b; Dugue and Mainen, 2009; Petzold et al., 2009; Liu et al., 2012; Schmidt and Strowbridge, 2014; Brill et al., 2016; Gaudry, 2018; Sizemore et al., 2020).

Similar to that of widespread serotoninergic innervation, centrifugal cholinergic fibers from the horizontal limb of the diagonal band of Broca innervate all layers of the olfactory bulb (Macrides et al., 1981; Záborszky et al., 1986). Bulbar neurons express nicotinic (nAChR) and muscarinic (mAChR) acetylcholine receptors (Castillo et al., 1999; Ghatpande et al., 2006; Pressler et al., 2007), conferring the cholinergic system the capacity to modulate specific synapses involved in olfactory information processing. At a circuit level, cholinergic modulation has been shown to target the glomerular microcircuit and modulate reciprocal, dendrodendritic synapses between MCs and GCs (Castillo et al., 1999; Ghatpande et al., 2006; Pressler et al., 2007). Behavioral studies indicate that the cholinergic system is involved in the regulation of several olfactory-guided behaviors in mice including odor discrimination (Doty et al., 1999; Chaudhury et al., 2009; D'Souza and Vijayaraghavan, 2014; Smith et al., 2015), short-term olfactory memory and fine tuning of MC activity (Devore and Linster, 2012), and olfactory perceptual learning (Fletcher and Wilson, 2002; Wilson et al., 2004).

All these neuromodulatory studies indicate that the increased excitatory drive in GCs might shift the excitatory/inhibitory (E/I) balance in MCs toward inhibition, ultimately influencing MCs input-output relationship. Therefore, acetylcholine (ACh) influences the final message to the olfactory cortex enhancing specificity and temporal precision of odor-evoked responses in MCs. Serotonergic and cholinergic modulation within the olfactory bulb is summarized in Table 2.

**Table 2:** Overview of centrifugal olfactory neuromodulators.

**Neuromodulator**	**Source**	**Receptor**	**Localization**	**Cellular effects**	**Behavioral effects**	**References**
Serotonin	Dorsal medial raphe nuclei innervate all layers of the olfactory bulb Dense dorsal projections to granule cell layer dense medial projections to glomerular layer	5-HT1A	Mitral cell layer, internal granular layer, external plexiform layer		Serotonin has a role in olfactory learning	McLean and Shipley, 1987a; Plassat et al., 1992; Pompeiano et al., 1992; Tecott et al., 1993; McLean et al., 1995, 1996; Yuan et al., 2003; Hardy et al., 2005b; D'Souza and Vijayaraghavan, 2012, 2014; Liu et al., 2012; D'Souza et al., 2013; Steinfeld et al., 2015; Kapoor et al., 2016; Huang et al., 2017
		5-HT2A	Mitral cell, tufted cell	Excite mitral cells and tufted cells, activate external tufted cell TRP channel-mediated cation current	Serotonin depletion prevents odor learning, recover with 5-HT2A/C agonist	
		5-HT2C	Juxtaglomerular cell	Depolarize juxtaglomerular cells		
		5-HT3	Glomerular layer (mRNA)			
		5-HT5	Tufted cell (mRNA)			
Acetylcholine	Horizontal limb of the diagonal band of Broca projects to all bulb layers, especially the internal plexiform layer and glomerular layer/external plexiform layer boundary	Nicotinic	Glomerular layer	nAChR activation excites mitral cells, periglomerular cells, and external tufted cells	Role in olfactory learning and discrimination	Macrides et al., 1981; Záborszky et al., 1986; Le Jeune et al., 1995; Castillo et al., 1999; Crespo et al., 2000; Fletcher and Wilson, 2002; Gómez et al., 2005; Pressler et al., 2007; Chaudhury et al., 2009; D'Souza and Vijayaraghavan, 2012, 2014; Pavesi et al., 2012; D'Souza et al., 2013; Smith et al., 2015; Ross et al., 2019
		Muscarinic	All bulb layers	mAChR activation decreases firing frequency of granule cells, increases transmitter release from granule cells onto mitral cells via dendro-dendritic synapses	Sharpen mitral cell odorant receptive fields	
		m1, m2	All bulb layers, especially external plexiform layer and granule cell	m1 mediates granule cell excitation m2 mediates granule cell inhibition	Olfactory fear learning involves mAChRs, requires m1	

*5-HT, serotonin; TRP, transient receptor potential; AChR, acetylcholine receptor*.

In summary, given the paucity of information regarding the purpose of this newly discovered microcircuit, we thereby focused our study upon what might modulate PPG neuron activity by testing suspected metabolic hormones or neurotransmitters well-studied in the olfactory bulb, and whose receptors were known to be expressed in this lamina. A series of *ex vivo* slice electrophysiology experiments were performed to determine the basal membrane properties of these neurons and identify possible changes in excitability induced by neurotransmitters or metabolic-related hormones that are common signaling molecules in the olfactory bulb. Less is known about how metabolic peptides and neuromodulators control specific neuronal subpopulations. Such a PGG>MC>GC microcircuit has the potential to be recruited to provide neuromodulation during ever changing metabolic states induced by feeding and fasting.

## Materials and Methods

### Ethical Approval

All animal experiments were approved by the Florida State University (FSU) Institutional Animal Care and Use Committee (IACUC) under protocol #1427 and were conducted in accordance with the American Veterinary Medicine Association (AVMA) and the National Institutes of Health (NIH). In preparation for olfactory slice electrophysiology, mice were anesthetized with isoflurane (Aerrane; Baxter, Deerfield, IL, USA) using the IACUC-approved drop method and were then sacrificed by decapitation (Leary, 2020).

### Animal Care

Detection of pre-proglucagon (PPG) neurons expressing a red fluorescent protein (RFP) was achieved by crossing Rosa26-tandem-dimer red fluorescent protein (tdRFP) reporter mice (Luche et al., 2007) with mice expressing Cre recombinase under the control of the pre-proglucagon promoter (GLU-Cre12 mice) (Parker et al., 2012). For simplification, homozygous progeny resulting from the breeding of GLU-Cre12 and Rosa26 tdRFP mice are referred to as PPG-Cre-RFP mice (Thiebaud et al., 2019). All mice were housed in the Florida State University vivarium on a standard 12 h/12 h light/dark cycle and were allowed *ad libitum* access to 5001 Purina Chow (Purina, Richmond, VA, USA) and water. Mice of both sexes at post-natal day 20–45 were used for slice electrophysiology experiments.

### Solutions and Reagents

Artificial cerebral spinal fluid (ACSF) contained (in mM): 119 NaCl, 26.2 NaHCO_3_, 2.5 KCl, 1 NaH_2_PO_4_, 1.3 MgCl_2_, 2.5 CaCl_2_, 22 glucose; 305–310 mOsm, pH 7.3-7.4. Sucrose-modified artificial cerebral spinal fluid (sucrose ACSF) contained (in mM): 83 NaCl, 26.2 NaHCO_3_, 1 NaH_2_PO_4_, 3.3 MgCl_2_, 0.5 CaCl_2_, 72 sucrose, 22 glucose, 5 sodium ascorbate, 2 thiourea, 3 sodium pyruvate; 315–325 mOsm, pH 7.3–7.4. The intracellular pipette solution contained (in mM): 135 K gluconate, 10 KCl, 10 HEPES, 10 MgCl_2_, 2 Na-ATP, 0.4 Na-GTP; 280–290 mOsm, pH 7.3–7.4. All salts and sugars were purchased from Sigma-Aldrich (St. Louis, MO, USA) or Fisher Scientific (Pittsburgh, PA, USA). The synaptic blockers 2,3-dihydroxy-6-nitro-7-sulfamoyl-benzo[f]quinoxaline (NBQX), D-(-)-2-amino-5-phosphonopentanoic acid (APV), and 2-(3-carboxypropyl)-3-amino-6-(4 methoxyphenyl) pyridazinium bromide (gabazine) were purchased from Ascent Scientific (Princeton, NJ, USA). All synaptic blockers were prepared as stock solutions (NBQX 5 mM, APV 25 mM, gabazine 6 mM) in Milli-Q water and stored at −20°C. They were diluted to working concentrations (NBQX 5 μM, APV 50 μM, gabazine 6 μM) in ACSF on the day of use. All pharmacological agents were introduced to the olfactory bulb slices through the bath chamber using ACSF as the control vehicle.

Serotonin hydrochloride (5-HT, H9523–100 mg, Sigma) was prepared at stock concentration (0.8 mM) in ACSF and was diluted to working concentrations (40 μM) in ACSF on the day of use. Stock solutions were prepared in Milli-Q water for the following drugs that were then diluted in ACSF to working concentrations on the day of use: 5 mM acetylcholine chloride (ACh, A6625-10 mg, Sigma), 0.2 mM cholecystokinin octapeptide (sulfated) ammonium salt (CCK, H2080-1 mg, Bachem Americas, Inc., Torrance, CA), 0.1 mM leptin (116–130) amide (mouse) trifluoroacetate salt (Leptin, H3966-1 mg, Bachem).

### Olfactory Bulb Slice Electrophysiology

Mice were anesthetized by inhalation of isoflurane (see Ethical Approval section), quickly decapitated, and then the olfactory bulbs were exposed by removing the dorsal and lateral portions of the skull between the lambda suture and the cribriform plate. The olfactory bulbs were harvested and prepared for slice electrophysiology as described previously (Fadool et al., 2011). Briefly, after removing the dura, a portion of forebrain attached with the olfactory bulbs were cut and quickly glued to a sectioning block with Superglue (Lowe's Home Improvement, USA), and submerged in oxygenated (95%O2 / 5%CO2), ice-cold, sucrose-modified ACSF for ~2 minutes (min) prior to vibratome sectioning (Vibratome/Leica Model 1000, Wetzlar, Germany). Coronal sections were made at a thickness of 300 μM and then allowed to recover in an interface chamber (Krimer and Goldman-Rakic, 1997) for 20–30 min at ~33°C containing oxygenated ACSF. The slices were then maintained at room temperature (~23°C) for about 60 min before recording. Olfactory bulb slices were recorded in a continuously-perfused (Ismatec; 1–2 ml/min), submerged-slice recording chamber (RC-26, Warner Instruments, Hamden, CT) with ACSF at room temperature. Slices were visualized at 10× and 40× using an Axioskop 2FS Plus microscope (Carl Zeiss Microimaging, Inc., Thornwood, NY) equipped with infrared detection capability (Dage MTI, CCD100, Michigan, IN). Electrodes were fabricated from borosilicate glass (Hilgenberg #1405002, Malsfeld, Germany) to a pipette resistance ranging from 9 to 15 MΩ. Positive pressure was retained while navigating through the olfactory bulb laminae until a slight increase in the pipette resistance (typically 0.1–0.2 MΩ) was observed; indicating that the pipette tip had made contact with the cell. A giga-ohm seal (Re = 2.0–16.4 GΩ) was achieved by releasing positive pressure and simultaneously applying a light suction. The whole-cell configuration was established by applying a rapid but strong suction to the lumen of the pipette while monitoring resistance.

After establishing a whole-cell configuration, PPG neurons were first sampled for adequate resting potential (< –70 mV) and proper series resistance (<60 MΩ) prior to initiating a series of current-clamp recordings. Perithreshold current levels were determined by incrementally injecting 1,200 milliseconds (ms)-long, 25 pA steps of current every 10 s, starting at −100 pA. Following the determination of spike threshold, cells were then stimulated with a long, perithreshold current step of 5,000 ms duration (typically ranging from 5 to 50 pA) every 18 s to acquire spike frequency data.

### Data Acquisition and Statistical Analysis

Current-clamp experiments were performed using a Multiclamp 700B amplifier (Axon Instruments, Molecular Devices, Sunnyvale, CA). The analog signal was filtered at 10 kHz and minimally digitally sampled every 100 μs. The signals were digitized with a Digidata 1440A digitizer (Axon Instruments, Molecular Devices). The pipette capacitance was electrically compensated through the capacitance neutralization circuit of the Multiclamp 700B amplifier. Resting membrane potentials were corrected for a calculated −14 mV junction potential offset. Membrane capacitance and input resistance were acquired from the membrane test function of Clampex 10.3 (Axon Instruments). Data were analyzed using Clampfit 10.3 (Axon CNS), in combination with the analysis packages Origin 8.0 (MicroCal Software, Northampton, MA), and Igor Pro 6.0.2 (Wavemetrics Inc., Portland, OR) with the NeuroMatics 2.02 plugin (written by Jason Rothman). Baseline, treatment, and washout values were calculated from the mean of at least 10 consecutive traces. Statistical significance was determined between baseline biophysical property and that following the modulator using a two-tailed, paired *t-*test or a one-way repeated measures (RM) analysis of variance (ANOVA) at the 95% confidence level (α = 0.05). All sampled populations were analyzed using Prism 6 (GraphPad Software Inc., CA, USA). All reported values are mean (standard deviation -SD) unless otherwise noted.

## Results

### Electrophysiological Properties of PPG Neurons

**Figure 3:**
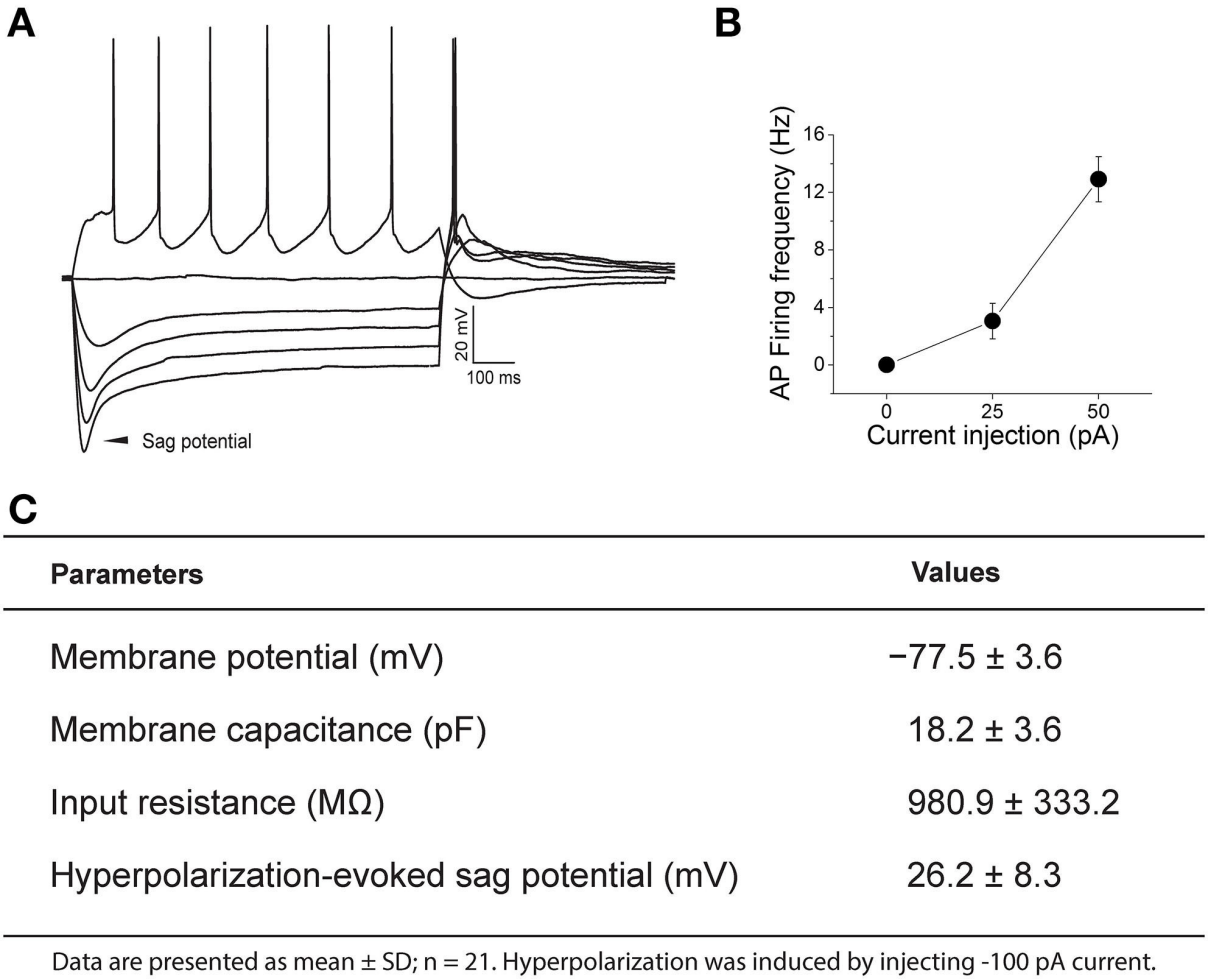
Representative current-clamp recording of a pre-proglucagon (PPG) neuron. **(A)** Perithreshold current levels were determined by incrementally injecting 1,200 ms-long, 25 pA steps of current every 10 s, starting at −100 pA. Notice the rebound firing after hyperpolarization and the degree of adaptation in action potential (AP) firing frequency. **(B)** Plot of injected current vs. mean action potential firing frequency (input-output relationship) for 21 sampled PPG neurons. **(C)** Basic intrinsic properties of a sampled population of PPG neurons are listed in the chart.

Under our recording conditions nearly all PPG neurons lacked spontaneous firing at rest. Once an adequate resting membrane potential (< −70 mV) was sampled, perithreshold current levels were determined by incrementally injecting 1,200 milliseconds (ms)-long, 25 pA steps of current every 10 s, starting at −100 pA (Figure 3). All PPG neurons showed a “sag” potential at a hyperpolarized state. The “sag” potential is associated with hyperpolarization-activated, cyclic nucleotide-gated (HCN) channels (He et al., 2014) and is defined as the membrane potential difference between the peak potential and the tail potential. Basic electrophysiological properties of PPG neurons are tabled for a population of 21 neurons along with a representative recording and summary graph of action potential firing frequency vs. injected current (input-output) in Figure 3.

### The Regulation of PPG Neurons by Centrifugal Projections

**Figure 4:**
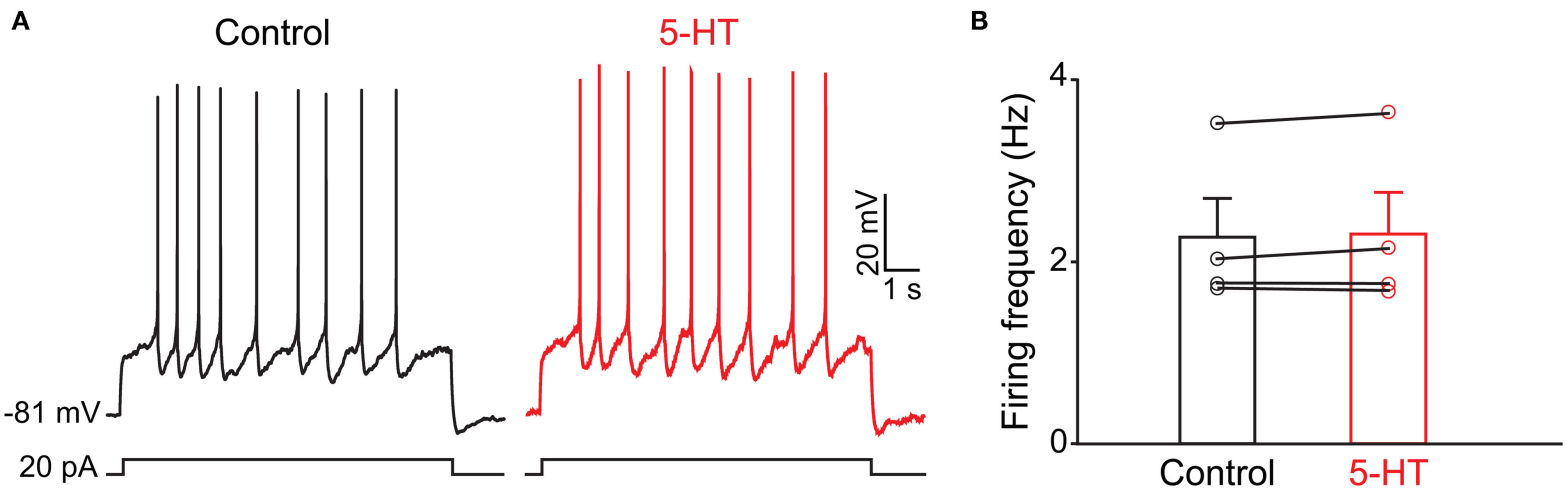
The activity of PPG neurons is not modulated by serotonin (5-HT) **(A)** Representative current-clamp recording elicited by injecting a perithreshold current of 25 pA with a pulse duration of 5 s in 18 s intervals. A baseline recording of 15 min was acquired before switching to bath application of 5-HT for an additional 30 min. **(B)** Bar/scatter plot of the mean AP firing frequency under baseline then 5-HT stimulation conditions in 4 cells, not significantly different, paired *t*-test, *p* > 0.05.

**Figure 5:**
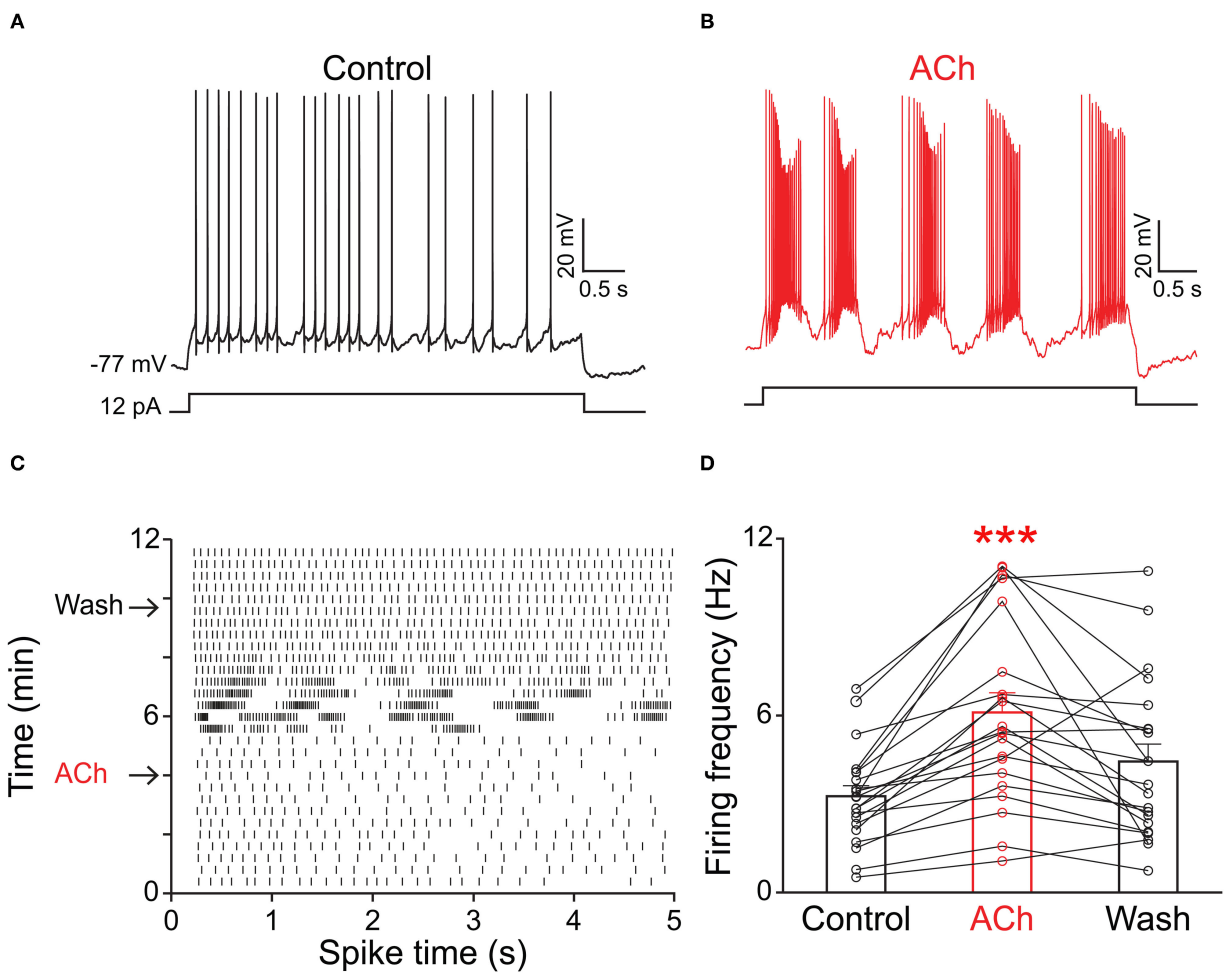
PPG neurons were excited by acetylcholine (ACh). Representative current-clamp recording elicited by injecting a perithreshold current of 12 pA with a pulse duration of 5 s in 18 s intervals. **(A)** A baseline recording of 5 min was acquired before **(B)** switching to bath application of ACh for 5 min, followed by a wash out. Note development of bursting activity with prominent spike adaptation within the burst. **(C)** Example raster plot of the cell in A-B indicating 5 s pulse duration vs. time of recording period (12 min). Arrows indicate the times when ACh was introduced or washed from the bath, respectively. **(D)** Bar/line graph of the mean spike frequency changes for 21 sampled PPG neurons under baseline, ACh, and wash conditions. ***Significantly-different from baseline, one-way RM ANOVA with Tukey's *post-hoc* test, *p* < 0.001.

Because the olfactory bulb receives multiple centrifugal projections from higher brain areas including serotonergic, cholinergic, and noradrenergic afferents, we first examined the possible top-down regulation of PPG neurons by these centrifugal projections. Despite widespread serotonin fiber innervation, bath application of serotonin (40 μM, *n* = 4) had no effect on PPG neuron evoked action potential firing frequency (Figures 4A,B, paired *t*-test, *p* > 0.05). Bath application of acetylcholine (ACh; 100 μM), however, caused increased excitation of PPG neurons (Figure 5). Recording in the current-clamp mode, bath application of ACh resulted in the development of a spike train with prominent spike adaptation over the course of the burst (Figures 5A,B). With continued ACh application, spike trains ceased over the course of 2–3 min and an increase in spike frequency remained (Figure 5C). The mean spike frequency was significantly increased (1.9 ± 0.6-fold; *n* = 21, 1-way RM ANOVA, Tukey's *post-hoc* test, *p* < 0.001, Figure 5D), compared with that of pre-stimulation and post-stimulation (wash). In an additional two cells, ACh delayed the latency to the first spike (control: 253 ± 30 ms, ACh: 396 ± 4 ms) but did not modify spike frequency.

### The Regulation of PPG Neurons by Metabolic-Related Signals

**Figure 6:**
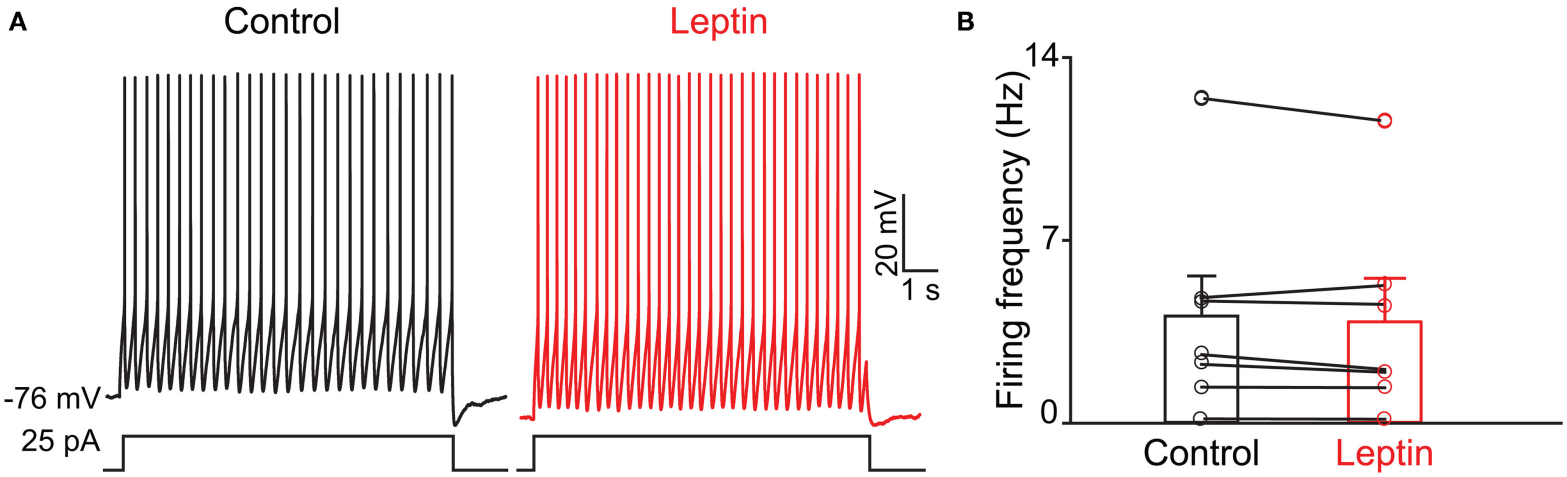
The activity of PPG neurons is not modulation by leptin. **(A)** Representative current-clamp recording and **(B)** bar/scatter plot of the mean AP firing frequency as in Figure 4, but for leptin, *n* = 7, not significantly different from baseline, paired *t*-test, *p* > 0.05.

**Figure 7:**
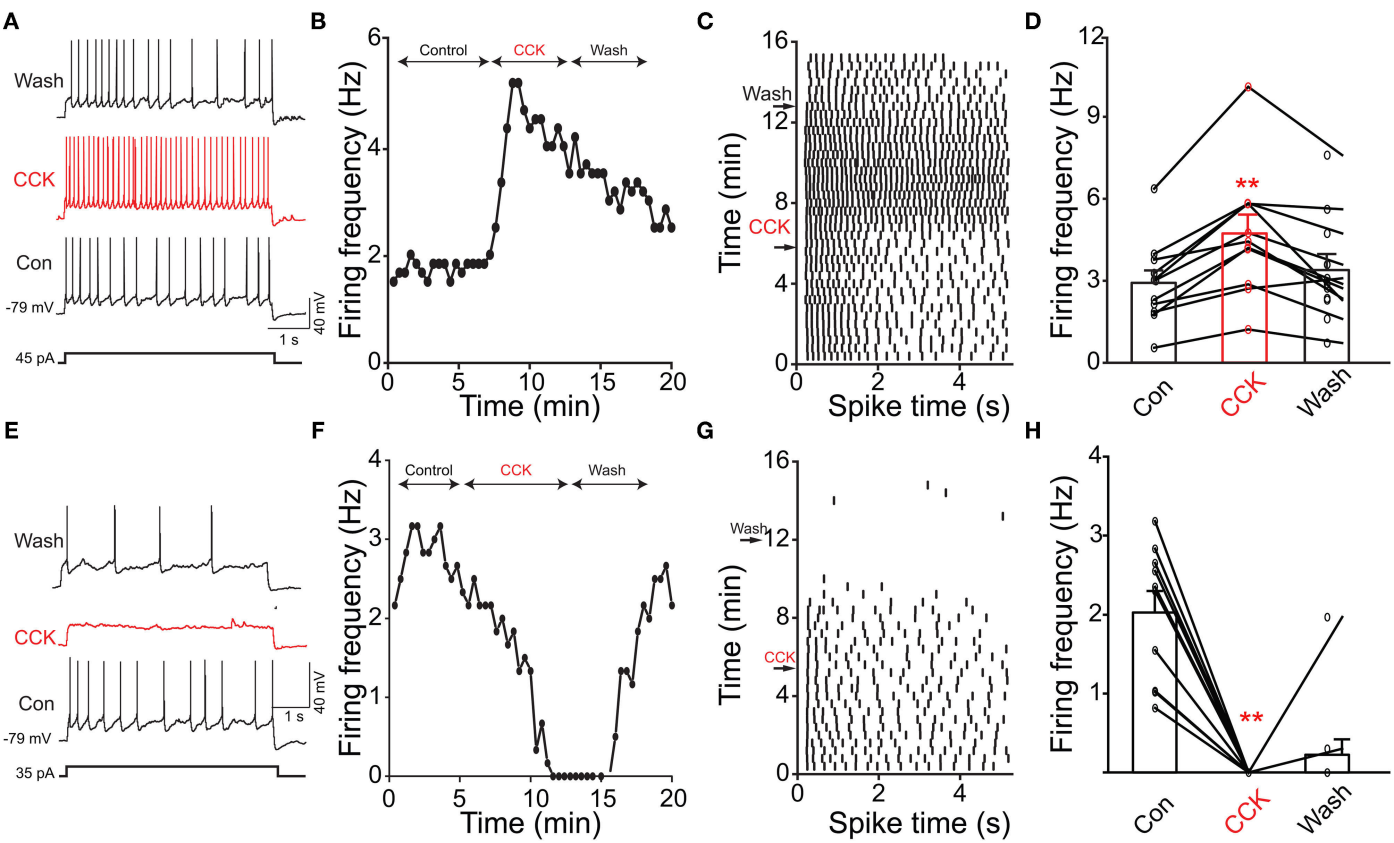
Cholecystokinin (CCK) evoked increased or decreased excitability of PPG neurons. **(A)** Representative action potentials elicited under baseline (Con), neuromodulator (CCK), and washout (Wash) conditions. **(B)** Line graph of the recording in A where AP firing frequency vs. time of the recording is plotted. **(C)** Example raster plot of the cell in **(A,B)**. **(D)** Bar/line graph of the mean spike frequency changes for 11 sampled PPG neurons. **Significantly-different from baseline and wash, one-way RM ANOVA with Tukey's *post-hoc* test, *p* < 0.01. **(E–H)** Same as top panels but for 10 sampled PPG neurons that were inhibited by CCK.

**Figure 8:**
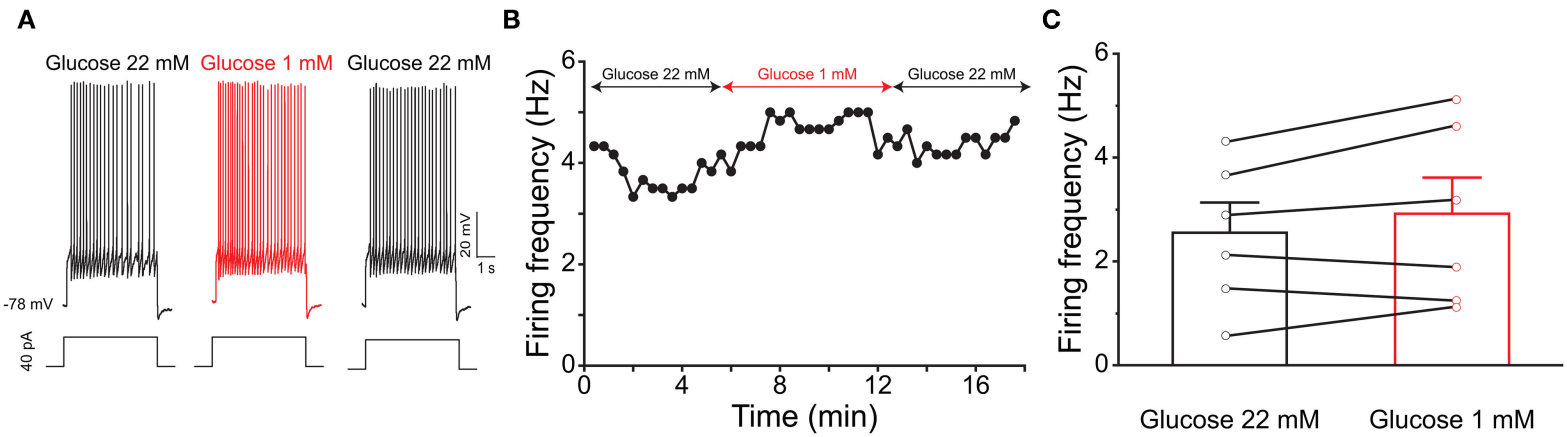
PPG neurons are not glucose sensitive. **(A)** Representative current-clamp recording elicited by injecting a perithreshold current of 40 pA with a pulse duration of 5 s in 18 s intervals. A baseline recording of 6 min was acquired for the PPG neuron under control ASCF conditions (22 mM) before switching to a bath application of low glucose (1 mM) for 6 min, followed by a return to original control ASCF for 6 min. **(B)** Line graph of the cell in (A) plotting action potential firing frequency over time. **(C)** Bar/line graph of the mean spike frequency changes for 6 of 16 sampled PPG neurons that had a change of at least 0.5 Hz following low glucose. Not significantly-different from baseline and wash, one-way RM ANOVA with Tukey's *post-hoc* test, *p* >0.05.

Previous evidence has shown that PPG neurons in the NTS can be modulated by metabolic-related hormones such as leptin or cholecystokinin (CCK) (Hisadome et al., 2010, 2011). Bath application of leptin did not significantly modulate action potential firing frequency of PPG neurons (Figures 6A,B, paired *t*-test, *p* > 0.05). Bath application of CCK (0.8 μM), however, led to either a significant increase in firing in 52 percent of the recorded neurons (1.7 ± 0.4-fold; *n* = 11, 1-way RM ANOVA, Tukey's *post-hoc* test, *p* < 0.01, Figures 7A–D) or cessation of firing (n = 10, Figures 7E–H) in 48% of the recorded neurons, where a majority of these inhibited neurons (8 of 10) did not recover following washout. Following the ingestion of a meal, another altered signal other than satiety hormones can be glucose availability. We were curious as to whether PPG neurons might be glucose sensitive as we previously reported for that of MCs (Tucker et al., 2013). PPG neurons were thus stimulated with a peri-stimulus evoked current intensity (40 pA) and then bath application of the standard ACSF (22 mM glucose) was switched to a modified ACSF balanced osmotically with mannitol (1 mM glucose). A subset of PPG neurons (6 of 16 cells; 38%) showed a modest increase in action potential firing frequency (1.2 ± 0.4-fold) that was not significantly different than that of baseline (paired *t*-test, *p* = 0.13) and was accompanied by a 1–2 mV depolarization (Figure 8).

## Discussion

**Figure 9:**
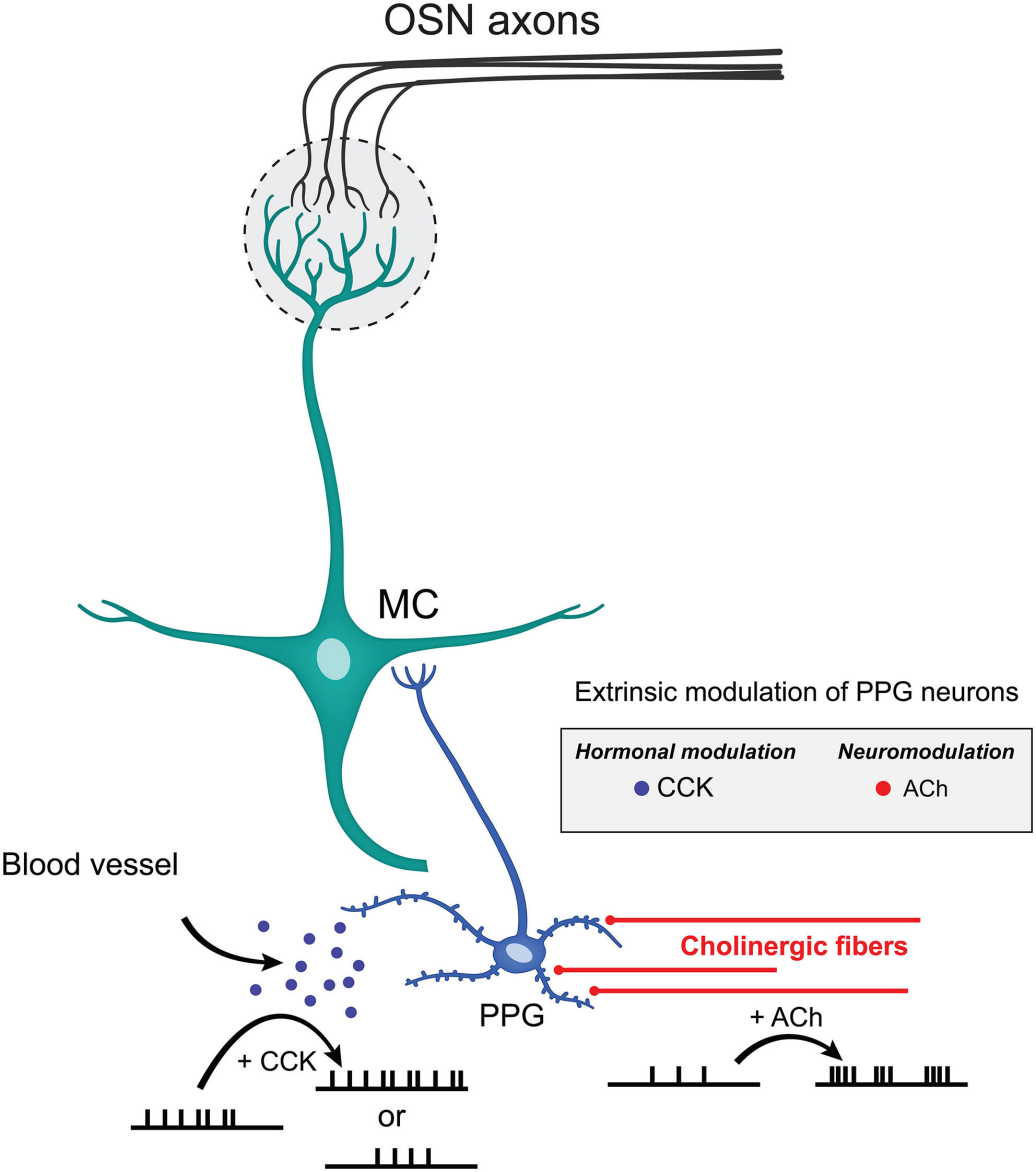
Extrinsic modulation of PPG neurons. Schematic diagram summarizing the effects mediated by CCK and ACh on PPG neuronal firing. CCK release by the blood vessels may either reduce or enhance AP firing frequency while ACh released by cholinergic fibers increases action potential firing frequency while eliciting bursting behavior. Such diversity of modulation of PPG neurons within a PPG>MC>GC microcircuit could allow great dynamics of outputted information by MCs to the higher olfactory cortical areas.

Performing *ex vivo* olfactory bulb slice experiments allowed us to understand the extent of neuromodulation of PPG neurons, a unique excitatory interneuron that is part of a recently discovered microcircuit. We discovered that these PPG neurons exhibit enhanced bursting and firing frequency in the presence of the neurotransmitter ACh yet are unmodulated by serotonin. Given that the olfactory bulb integrates both intrinsic and extrinsic regulatory feedback to shape MC and TC excitability before conveying the olfactory information to the piriform cortex (Cleland and Linster, 2005; Devore and Linster, 2012; Igarashi et al., 2012; Linster and Cleland, 2016; Lizbinski and Dacks, 2017), it appears that extrinsic sources of modulation that have been richly studied in the olfactory system (McLean and Shipley, 1987a,b; Mandairon et al., 2006; Matsutani and Yamamoto, 2008; Fletcher and Chen, 2010; Devore and Linster, 2012; Lizbinski and Dacks, 2017; Brunert and Rothermel, 2021) could significantly impact the function of PPG neurons (Figure 9). Extrinsic neuromodulation is thought to provide contextual information regarding the behavioral and chemical state of an animal and to influence olfactory sensitivity and olfactory-based behaviors. We also found that these PPG neurons could be differentially modulated by the metabolic-related hormone CCK but were not responsive to leptin. Metabolic peptides, neuropeptides, and hormones represent an extra source of extrinsic modulation in the olfactory system (Palouzier-Paulignan et al., 2012).

In defining the intrinsic properties of PPG neurons, the resting potential was more negative than that of GCs in general, but the input resistance was very much in keeping with values reported for GCs (Wellis and Kauer, 1994; Hall and Delaney, 2002; Pinato and Midtgaard, 2003). All PPG neurons exhibited a significant hyperpolarization-induced “sag” potential. The “sag” potential is attributed to a hyperpolarization-activated, cyclic nucleotide-gated (HCN) channel (He et al., 2014). In mammals there are four subtypes of this channel (HCN1-4) expressed widely throughout the CNS (Notomi and Shigemoto, 2004). Although all isoforms have been immunocytochemically identified in the olfactory bulb, HCN3-expressing axon bundles have been noted to be dispersed across the GCL (Notomi and Shigemoto, 2004). Activation of HCN channels leads to increased permeability of potassium and sodium ions, producing an inward, *I*_*h*_ current (Biel et al., 2009). *I*_*h*_ current is known to play important roles in stabilizing the resting membrane potential (Llinas and Jahnsen, 1982; Lupica et al., 2001) and integrating the synaptic inputs (Magee, 1998). *I*_*h*_ current has been implicated in a variety of physiological processes including learning and memory, sleep and wakefulness, sensation, and perception (Robinson and Siegelbaum, 2003). It has been shown that *I*_*h*_ currents are involved in adjusting sensory signal transduction and perceiving environmental stimuli (Orio et al., 2009; Zhou et al., 2010). In the visual system, *I*_*h*_ current has been well-characterized in photoreceptor cells where it has been shown to contribute to visual adaptation to bright light (Bader et al., 1979; Attwell and Wilson, 1980). In the taste system, HCN channels generate the sensory receptor potential to mediate sour taste response (Stevens et al., 2001). In general, HCN channels are regulated by wide-ranging cellular signals and their dysregulation has been shown to involve multiple pathological conditions such as epilepsy, neuropathic pain, parkinsonian disease (He et al., 2014). Interestingly ACh can both inhibit (Heys et al., 2010) and upregulate (Pian et al., 2007) HCN channels. It will be interesting to examine whether the modulation of PPG neurons by ACh is targeting *I*_*h*_ current, which may adjust olfactory signal transduction and eventually lead to changes in the olfactory perception.

The important role of cholinergic modulation of olfactory acuity has been long established (Fletcher and Wilson, 2002; Wilson et al., 2004; Chaudhury et al., 2009; Devore et al., 2014; D'Souza and Vijayaraghavan, 2014; Smith et al., 2015; Linster and Cleland, 2016; Cho and Linster, 2020). More specifically, odor response tuning of M/TCs is sharpened by the cholinergic input, thereby facilitating contrast enhancement (Castillo et al., 1999; Ma and Luo, 2012). The olfactory bulb receives cholinergic input from the horizontal limb of the diagonal band of Broca (HDB) of the basal forebrain (Záborszky et al., 1986; Kasa et al., 1995). Interestingly, this basal forebrain cholinergic system also projects to the hypothalamus and has been shown to modulate appetite-related synapses in lateral hypothalamic slices (Jo et al., 2005). A recent study has shown that the basal forebrain to hypothalamus cholinergic circuit plays an important role in regulating feeding behavior (Herman et al., 2016). When the cholinergic signaling was impaired either by ablating cholinergic neurons or knockdown of the transmitter's degradation enzyme, acetylcholine transferase, animals showed increased food intake leading to severe obesity. Alternatively, enhanced cholinergic signaling led to decreased food intake. Analogous to these studies, a link between satiation/positive energy state and altered olfactory processes could be constructed. Through unknown mechanisms, feeding activates the basal forebrain cholinergic neurons (Herman et al., 2016), which, in turn, will act on hypothalamic targets to exert their appetite suppression effect. One could speculate that this could simultaneously modulate the activity of PPG neurons in the olfactory bulb to alter olfactory processes.

Hormones and nutritionally important molecules that govern our state of satiety and hunger are classically defined as either orexigenic or anorexigenic signals, meaning those that stimulate or inhibit food intake, respectively. These molecules are produced by the gastrointestinal tract, adipose tissue, and the pancreas, and serve as an additional source of extrinsic modulation to the olfactory system, and, in particular, the olfactory bulb (Palouzier-Paulignan et al., 2012; Julliard et al., 2017; Kolling and Fadool, 2020). Receptors for orexigenic signaling pathways (i.e., ghrelin, neuropeptide Y, endocannabinoids, orexin, somatostatin) and anorexigenic pathways (i.e., insulin, GLP-1, leptin, and CCK) are expressed throughout the neurolamina of the olfactory bulb [see detailed reviews - Palouzier-Paulignan et al. (2012) and Julliard et al. (2017)].

Leptin and CCK are two anorectic hormones that we examined as neuromodulators of PPG neurons. Both are synthesized in the periphery and curb hunger. Removal of leptin or leptin receptors in mice causes an increase in olfactory performance in hidden odor tasks, which is decreased to control levels when the hormone is restored (Getchell et al., 2006). Central injections of leptin into fasted rats causes a dose-dependent decrease in olfactory detection (Julliard et al., 2007). Leptin receptors are found in the GML and on MCs (Shioda et al., 1998; Prud'homme et al., 2009), and also on astrocytes within the GCL, rather than on neurons (Prud'homme et al., 2009). This astrocytic pattern of GCL expression of the hormone receptor may be consistent with our lack of direct modulation of the PPG neurons in this lamina. In contrast, PPG neurons had differential responses to CCK: some neurons were excited, and some were inhibited. Such a heterogeneous response might suggest different subtypes of PPG neurons that express different CCK receptors or different activation of downstream intracellular signaling pathways. As a whole, few functional studies have examined CCK modulation in the olfactory bulb. *Ex vivo* recordings have indicated that CCK modulates MCs excitability by increasing action potential frequency (Ma et al., 2013) and behavioral studies have shown that activation of CCK receptors (CCK-A and CCK-B) modulate olfactory recognition and memory retention in rodents (Lemaire et al., 1994a,b).

Finally, the blood brain barrier surrounding the olfactory bulb is more permeable than other brain regions (Ueno et al., 1991, 1996) and it has been suggested that metabolic molecules can easily penetrate and bind to receptors for hormones broadly expressed in the olfactory system to modulate the electrical activity of olfactory networks (Fadool et al., 2000, 2011; Apelbaum et al., 2005; Hardy et al., 2005a; Lacroix et al., 2008; Savigner et al., 2009; Kuczewski et al., 2014). For example, insulin and glucose modulate the firing activity of MCs through post-translational modifications and other interactions with the voltage-gated potassium ion channel, Kv1.3 (Fadool et al., 2000, 2011; Savigner et al., 2009; Kuczewski et al., 2014). Despite this, we did not observe any significant glucose sensitivity of PPG neurons under our recording conditions. It may be that a combined environment where there are changes in both neurotransmission and metabolic factors, is required to produce synergistic changes for modulation of PPG neuronal excitability. It would be interesting in future investigations to explore cholinergic modulation, for example, while modifying glucose availability.

In summary, our study has furthered our biophysical understanding of a novel class of dSACs called PPG neurons that define a microcircuit within the olfactory bulb to modulate MC outputs. Future experiments need to probe olfactory behavioral changes in response to loss or gain of PPG neuron function. Because both central and peripheral effects of GLP-1 have demonstrated reduction in food intake (Williams, 2009), links between olfactory and ingestive behaviors should be sought. Due to the fact that GLP-1 is secreted after meal ingestion, it's possible that the GLP-1 system in the olfactory bulb could link weaker odor sensing to satiety state to inhibit food intake.

## Data Availability Statement

The original contributions presented in the study are included in the article/supplementary material, further inquiries can be directed to the corresponding author.

## Author Contributions

DF, RT, AL, and ZH: conceptualization. ZH: electrophysiological data collection and analysis. RT and DL: figure preparation. AL and RT: lead citation research and compiling. ZH, AL, RT, and DL: writing an original draft section. DF: writing—review and editing, supervision, project administration, and funding acquisition. All authors contributed to the article, written revisions, and approval of the submitted version.

## Conflict of Interest

The authors declare that the research was conducted in the absence of any commercial or financial relationships that could be construed as a potential conflict of interest.

## Abbreviations

Ach: acetylcholine
AVMA: American Veterinary Medicine Association
ANOVA: analysis of variance
AON: anterior olfactory nucleus
aPCx: anterior piriform cortex
ACSF: artificial cerebrospinal fluid
CCK: cholecystokinin
cAMP: cyclic adenosine monophosphate
dSAC: deep short axon cell
EPL: external plexiform layer
EPLi: external plexiform layer interneuron
ETC: external tufted cell
FSU: Florida State University
GPCR: G-protein-coupled receptor
GML: glomerular layer
GLP-1: glucagon-like peptide-1
GC: granule cell
GCL: granule cell layer
HDB: horizontal limb of the diagonal band of Broca
HCN: hyperpolarization-activated, cyclic nucleotide-gated ion channel
IACUC: Institutional Animal Care and Use Committee
IPL: internal plexiform layer
JG: juxtaglomerular
MC: mitral cell
MCL: mitral cell layer
M/TC: mitral/tufted cell
mAChR: muscarinic acetylcholine receptor
NIH: National Institute of Health
nAChR: nicotinic acetylcholine receptor
NTS: nucleus tractus solitarius
OB: olfactory bulb
OSN: olfactory sensory neuron
PG: periglomerular
PGC: periglomerular cell
PPG: pre-pro-glucagon
RFP: red fluorescent protein
RM: repeated measures
SAC: short axon cell
sSAC: superficial short axon cell
STC: superficial tufted cell
SNARE: soluble NSF attachment protein receptor
sTC: superficial tufted cell
tdRFP: tandem-dimer red fluorescent protein
TC: tufted cell.
